# SARS-CoV-2 and Multiple Sclerosis: Potential for Disease Exacerbation

**DOI:** 10.3389/fimmu.2022.871276

**Published:** 2022-04-22

**Authors:** Madison MacDougall, Jad El-Hajj Sleiman, Philippe Beauchemin, Manu Rangachari

**Affiliations:** ^1^ Department of Biological Sciences, Salisbury University, Salisbury, MD, United States; ^2^ Department of Psychology, Salisbury University, Salisbury, MD, United States; ^3^ Division of Neurology, Department of Medicine, CHU de Québec – Université Laval, Quebec City, QC, Canada; ^4^ Axe Neurosciences, Centre de Recherche du CHU de Québec – Université Laval, Quebec City, QC, Canada; ^5^ Department of Molecular Medicine, Faculty of Medicine, Université Laval, Quebec City, QC, Canada

**Keywords:** COVID-19, SARS-CoV-2, multiple sclerosis, experimental autoimmune encephalomyelitis, neuroinflammation, blood-brain barrier, adaptive immunity, cytokine storm

## Abstract

While the respiratory tract is the primary route of entry for SARS-CoV-2, evidence shows that the virus also impacts the central nervous system. Intriguingly, case reports have documented SARS-CoV-2 patients presenting with demyelinating lesions in the brain, spinal cord, and optic nerve, suggesting possible implications in neuroimmune disorders such as multiple sclerosis (MS) and other related neuroimmune disorders. However, the cellular mechanisms underpinning these observations remain poorly defined. The goal of this paper was to review the literature to date regarding possible links between SARS-CoV-2 infection and neuroimmune demyelinating diseases such as MS and its related disorders, with the aim of positing a hypothesis for disease exacerbation. The literature suggests that SARS-CoV, SARS-CoV-2, and orthologous murine coronaviruses invade the CNS *via* the olfactory bulb, spreading to connected structures *via* retrograde transport. We hypothesize that a glial inflammatory response may contribute to damaged oligodendrocytes and blood brain barrier (BBB) breakdown, allowing a second route for CNS invasion and lymphocyte infiltration. Potential for molecular mimicry and the stimulation of autoreactive T cells against myelin is also described. It is imperative that further studies on SARS-CoV-2 neuroinvasion address the adverse effects of the virus on myelin and exacerbation of MS symptoms, as nearly 3 million people suffer from MS worldwide.

## Introduction

Multiple sclerosis (MS) is a disease that affects over 2.8 million people worldwide, causing chronic neuroinflammation and neurodegeneration of CNS myelin (1). Despite its high prevalence, the etiology of MS is not fully understood. Epstein Barr Virus (EBV) seropositivity is strongly linked to MS incidence (2, 3) and a recent study provided compelling evidence of a causative role for EBV in MS development (4). This viral hypothesis, coupled with the current COVID-19 pandemic, poses the question of whether SARS-CoV-2 infection might exacerbate MS.

There have been case reports linking COVID-19 and demyelination in mouse and man (5–7), as well as retrospective studies showing a link between COVID-19 infection, MS symptom exacerbation, and relapse (8, 9). However, the mechanisms of these processes have not been elucidated. With the long-term consequences of SARS-CoV-2 infection still unclear, it is important that we examine its potential impact on MS, including its ability to trigger demyelination and stimulate an inflammatory microenvironment that can worsen MS symptoms. Here we propose a model for SARS-CoV-2-triggered MS exacerbation, tracing a possible route of neuroinvasion and examining the role of glial cells, BBB, and molecular mimicry in the process.

## SARS-CoV-2

### Coronaviruses

Coronaviruses are enveloped RNA viruses belonging to the family *Coronaviridae*. A handful of coronaviruses are pathogenic to humans and cause respiratory complications (10). Severe acute respiratory coronavirus (SARS-CoV) and Middle East respiratory syndrome coronavirus (MERS-CoV) are associated with severe pneumonia, and they triggered epidemics in 2002/2003 and 2012, respectively. SARS-CoV infections dwindled in 2004, leading researchers to speculate that the virus was circulating among animals while raising the possibility of another animal-human transmission of a related virus in the future (10).

In December 2019, a novel betacoronavirus with 79.6% sequence homology to SARS-CoV was reported in Wuhan, China. Named severe acute respiratory coronavirus-2 (SARS-CoV2), it quickly sparked the worldwide coronavirus disease 19 (COVID-19) pandemic (11–13). Symptoms of COVID-19 include fever, anosmia, headache, and dry cough, as well as dyspnea, pneumonia, and respiratory failure due to damaged alveoli (11, 12, 14). Similar lung pathology is recapitulated in murine models of SARS-CoV-2, including viral presence in the lungs, interstitial pneumonia, hyaline membrane formation, edema, blocked terminal bronchioles, and monocyte and macrophage infiltration into alveolar spaces (15, 16). Similar to SARS-CoV, SARS-CoV2 utilizes Spike proteins to gain access to cells (17). While the Spike1 (S1) protein binds to human angiotensin converting enzyme 2 (ACE2), Spike2 (S2) is responsible for membrane fusion, which is made possible through protein cleavage by the protease TMPRSS2 (17, 18). Pulmonary complications are not surprising, given the high ACE2 expression in alveolar epithelial cells (19). However, ACE2 expression has also been observed in enterocytes of the small intestine, pericytes of the heart, and endothelial cells in various organs, including the brain (19, 20). Interestingly, recent studies show ACE2 expression in neurons of numerous brain structures, as well as in glial cells such as astrocytes and oligodendrocytes, suggesting possible implications of viral infection on the central nervous system (CNS) (21).

### COVID-19 and Nervous System Pathology

Indeed, while respiratory complications are a hallmark of COVID-19, evidence of viral neurotropism and neurological complications have emerged. For example, many COVID-19 patients present with symptoms such as anosmia, ageusia, brain fog, and cognitive disturbances (22–24). A rising number of COVID-19 “long haulers” with persistent neurocognitive and memory symptoms also suggest neurological involvement (25). After the initial acute phase of COVID-19 is resolved, some patients continue to develop post infectious symptoms that cannot be attributed to another disease. These patients have long COVID (if the symptoms last from 4 weeks to 12 weeks after the initial acute phase) or post-COVID-19 syndrome (if the symptoms persist after 12 weeks). Symptoms of these affections range from persistent chest pain and dyspnea to coagulopathy, fatigue, and cognitive changes (26). The pathophysiology of long COVID is poorly understood, but the increasing evidence of neurological symptoms poses the question of whether SARS-CoV-2 infection can exacerbate underlying neurological conditions, and whether this exacerbation is caused by primary damage from the virus itself or by secondary damage from an inflammatory cascade. As viral presence can trigger increased expression of IL-6, TNF-α, and other proinflammatory cytokines in a “cytokine storm,” understanding how this inflammatory cascade damages nervous system structures during SARS-CoV-2 infection is essential. This is especially true in the context of neurological disorders with an autoimmune basis such as multiple sclerosis (MS), as these individuals already have elevated cytokine levels and are frequently prescribed disease modifying therapies (DMTs) that can weaken their immune systems (27). In fact, some researchers found that COVID-19 patients with MS taking the B-cell-depleting reagents rituximab or ocrelizumab may be at an increased risk of hospitalization, ICU admission, and artificial ventilation (28). With over 490 million cases of COVID-19 and greater than 6 million deaths worldwide as of April 2022 (29), it is imperative to understand if those with MS are at an increased risk.

### Immune Responses in COVID-19

SARS-CoV-2 enters the body through the respiratory route and gains access to epithelial cells in the nasal cavity where it replicates (12, 30). At this stage, patients are already infectious, despite being typically asymptomatic (31). As the virus progresses through the respiratory tract, the innate immune response becomes more potent, and patients become symptomatic. This limited presentation, largely restricted to the upper airways, is what most patients will develop. For the remainder (~20%), however, the virus will continue its route to reach the alveoli and subsequently infect alveolar type II cells, leading to their apoptosis and loss of surfactant (31, 32). Throughout this manuscript, we refer to the virus itself as “SARS-CoV-2” and the resulting pathology as “COVID-19”.

Unsurprisingly, the immune response to COVID-19 mirrors that of other viral infections. Some elements of its virology have not been fully studied but can be extrapolated from findings on the SARS-CoV and MERS-CoV viruses. Adaptive immunity to the virus is triggered by viral antigen presentation by antigen-presenting cells (APC) in the context of major histocompatibility complex (MHC) molecules (33). Activated cytotoxic T lymphocytes (CD8^+^) are able to directly kill infected viral cells. In SARS-CoV infected patients, CD8^+^ T cells account for 80% of total infiltrative inflammatory cells in the pulmonary interstitium, where they clear infected cells and induce immune injury (34). Activated helper T cells (CD4^+^) play a critical role in the humoral response, as they activate T cell-dependent B cells to produce specific IgG antibodies against the virus. They also help coordinate the body’s response to the attack by secreting cytokines and chemokines to recruit different immune effector cell populations to the site of infection. An exaggerated immune response can however lead to a deadly systemic inflammatory response called “cytokine storm” (32–34).

## Multiple Sclerosis

Multiple sclerosis is a chronic autoimmune disease of the CNS that is characterized by demyelination, multifocal inflammation, and neuronal loss. MS leads to numerous motor and sensory deficits such as decreased mobility, impaired dexterity, vision loss, and bladder dysfunction (35). As MS onset often occurs between 20 and 40 years of age (36), MS-affected individuals can face decades of disability. MS has traditionally been considered a primarily CD4^+^ T cell mediated disorder; however, it is now clear that other adaptive and innate immune cells play critical roles in its pathogenesis.

MS is defined by clinical episodes of neurological relapses disseminated over time and by evidence of neuroanatomical lesions disseminated in space (37). It is usually diagnosed in the presence of typical clinical features, white-matter lesions on the MRI, and oligoclonal bands in the cerebrospinal fluid (CSF). Classic manifestations may include unilateral optic neuritis (ON), partial transverse myelitis (TM), and focal brainstem syndrome (37). The first clinical attack of MS is referred to as a clinically isolated syndrome (CIS), unless multiple white-matter lesions are found on the MRI. Up to half of patients with a ON CIS will subsequently develop MS (38). Over the course of the next two decades, 30-60% of patients with the relapsing-remitting (RRMS) subtype will develop a secondary progressive (SPMS) form associated with impaired cognition and progressive impairment of ambulation (39). In some cases, patients present with a primary progressive disease from the onset (40). Over 2.8 million people suffer from MS worldwide, yet treatment is limited to reducing inflammation and relapses, and there is currently no cure for MS (1).

### Other Neuroimmune Disorders

Neuromyelitis Optica Spectrum Disorder (NMOSD) is an antibody-mediated demyelinating disease of the CNS that can share similarities with RRMS at presentation. Like MS, it often presents as ON or TM, albeit with myelitis that is usually more extensive and severe than the partial myelitis seen in MS (41). A progressive course is uncommon. Diagnosis is usually made in the presence of a classical clinical syndrome when aquaporin (AQP)-4 antibodies are positive. AQP4 is abundant in the brain and the spinal cord, localizing to astrocytic membranes at the blood-brain barrier (42). AQP4-positive NMOSD represents two-thirds of cases (43). Seronegative subtypes do exist, but whether they are caused by an unknown antibody is a matter of debate.

Interleukin-6 (IL-6) has a key role in the pathophysiology of NMOSD (44). IL-6 promotes the differentiation of naïve T cells into pro-inflammatory Th17 and enhances the differentiation of B cells into plasmablasts that produce AQP4 antibodies (45). Further, it is a pro-inflammatory cytokine involved in the COVID-19 cytokine storm (46). NMOSD therapies, directed at IL-6 blockade, are shown to be effective (47).

Myelin oligodendrocyte glycoprotein (MOG) antibody disease (MOG-AD) is another neuroinflammatory condition that can mimic MS. It is also more prevalent in women, and typically presents as an ON in the majority of cases (48, 49). Other presenting features include myelitis and acute disseminated encephalomyelitis (ADEM) or ADEM-like presentations. Unlike MS however, oligoclonal bands are usually absent from CSF, and MOG antibody IgG are detected in the serum. Interestingly, two cases of MOG-AD following SARS-CoV-2 have been published. Both patients presented with bilateral ON following COVID-19 infection and were AQP4 negative and MOG positive (50, 51).

### Adaptive Immune Mechanisms in MS

Myelin reactive helper (CD4^+^) and cytotoxic (CD8^+^) T cells play important roles in the pathophysiology of MS and are present in MS lesions (40). In healthy individuals, CD4^+^ T cells help coordinate the body’s immune response to specific microbes. When presented with their cognate antigen in peripheral lymphoid tissues such as the spleen or lymph nodes, naïve T cells become activated and subsequently differentiate into subsets that release specific cytokines to help recruit and activate other leukocytes. In MS, it is postulated that peripherally activated CNS antigen-specific CD4^+^ T cells are locally reactivated in the CNS, leading to cytokine release and inflammatory lesions (52, 53). Intriguingly, Th1 and Th17 CD4^+^ T cell effector subsets have both been linked to MS disease onset and progression and described as potential cytokine storm drivers (54, 55).

In the past decade, evidence has accumulated that B cells are also crucial players in MS pathogenesis, with the advent of anti-CD20 depleting therapies such as ocrelizumab (56). Intriguingly, CD20 is not expressed on antibody-secreting plasma B cells, indicating that B cells may contribute to MS processes through mechanisms that are independent of their ability to generate antibodies. Of note, B cells may be involved in the pathogenesis of cytokine storm in the context of viral infections (55).

### Role of the BBB in MS

Given the central role of peripheral immune cells in eliciting CNS damage, it is unsurprising that defects in BBB integrity are linked to MS pathology. The BBB is a complex structure that regulates molecular and cellular entry in the CNS. It is composed of microvascular endothelial cells held together by tight junctions, glial cell elements, astrocytes, microglia, and a basement membrane (57, 58). Its purpose is to maintain hemostasis, allow for proper neuronal function, and shield neural tissue from toxins, microbes, and inflammation (59). In EAE models and in perivenous MS lesions, BBB disruption and microglial activation are the first pathological findings observed at disease onset within the CNS (60). They appear prior to lymphocyte infiltration and demyelination (61), though they are themselves preceded by an increase in the frequency of Th1 and Th17 cells in the immune periphery. An up-regulation of cell adhesion molecules on endothelial cells and a redistribution of junction proteins is also observed. This is associated with perivascular infiltration, as immune cells are found between the basement membrane and the endothelial cells, suggesting that these processes facilitate leukocyte migration into the CNS. Astrogliosis is linked to BBB leakage, and molecules produced by glial cells help increase BBB permeability (57).

## COVID-19 and Its Potential Role in MS Exacerbation

Recent case studies have reported demyelinating lesions in the brain, spinal cord, and optic nerve of COVID-19 patients. Acute TM, acute disseminated encephalomyelitis (ADEM), acute hemorrhagic leukoencephalitis (AHLE), and cytotoxic lesion of the corpus callosum (CLOCC) have been associated with SARS-CoV-2 infection (62). In some cases, patients with demyelinating lesions meet the diagnostic criteria for MS (**Table 1**), and cytokines involved in MS are upregulated in COVID-19 patients, suggesting a possible link between the virus and inflammatory CNS damage (5–7, 68, 69). A cohort study utilizing a questionnaire also revealed that out of 404 respondents with COVID-19 and MS, 51% had worsened pre-existing symptoms, and 20% developed new symptoms (70). Retrospective studies support these findings, one of which showed that 61% of MS patients with COVID-19 developed exacerbated symptoms, mainly motor and sensory issue exacerbations that occurred in the acute phase of COVID-19 (8). Another retrospective study of 41 RRMS patients with COVID-19 showed a significant increase in relapse during the at-risk period (ARP) of COVID-19, defined as two weeks before until five weeks after disease onset (9). In contrast, Etemadifar and colleagues did not find any long-term increase or exacerbation in RRMS patients’ clinical disease activity following COVID-19 (71). Despite increasing evidence for a potential impact of COVID-19 on MS, the mechanisms behind viral neurotropism, neuronal spread, and damage to myelin require further elucidation. This review offers a hypothesis of SARS-CoV-2 CNS infection and MS exacerbation. We posit that the direct CNS infection through the olfactory pathway can lead to gliosis and weakening of the BBB. At the same time, peripherally-generated immune responses can further potentiate neuroinflammation *via* adaptive immune molecular mimicry paired with cytokine storm. Given the rapidly moving state of the field, we considered both published papers, as well as manuscripts uploaded to preprint servers that have not yet undergone peer review.

**Table 1:** Case reports of MS following SARS-CoV-2 infection [adapted from (63)].

Author	Sex, Age	Time to symptoms after SARS-CoV-2 infection	Clinal manifestation	Laboratory testing	MRI findings	Diagnostic
Palao et al. (5)	F29	2-3 weeks	ON	Oligoclonal bands in CSF. Negative serum anti-IgG-NMO-AQP4 and anti-MOG antibodies.	Right-sided optic nerve lesion and supratentorial periventricular demyelinating lesions, with one gadolinium-enhancing lesions	MS
Yavari et al. (64)	F24	N/A	Diplopia, blurry vision, paresthesia, paresia	N/A	Multiple lesions in different brain areas	MS
Ismail et al. (65)	M36	2 months	Ataxia	Oligoclonal bands in CSF.	Multiple hyperintense lesions in both juxta-cortical and periventricular regions, as well as the cerebellum, with no contrast-enhancement	MS
Moore et al. (66)	M28	2 weeks	Paresthesia, internuclear ophthalmoplegia	5 unique oligoclonal bands in CSF. Negative CSF SARS-CoV-2. Negative serum AQP-4 and MOG.	Contrast-enhancing and non-enhancing white matter lesions in juxtacortical, periventricular and infratentorial locations.	MS
Zanin et al. (6)	F54	N/A	Unconsciousness	Negative CSF RT-PCR for SARS-CoV-2. Normal CSF examination.	Periventricular hyperintense white matter alteration, without contrast enhancement. Similar lesions present at the bulbo-medullary junction and in both the cervical and dorsal spinal cord.	MS
Karsidag et al. (67)	F42	1 month	Jaw and facial pain, numbness. Five months after the first attack, new weakness in legs and paresthesia with bladder incontinence.	Negative oligoclonal bands in CSF. Negative AQP4-IgG. Negative CSF SARS-CoV-2 PCR.	Multiple bilateral periventricular hyperintense lesions, some showing contrast enhancement and a single contrast-enhancing hyperintense lesion covering 1 segment on cervical MRI.	MS
Karsidag et al. (67)	M32	4 months	Jaw numbness After two months, new weakness in the right leg.	Type II oligoclonal bands in CSF. Positive CSF PCR for SARS-CoV-2.	Periventricular hyperintense lesions, some showing contrast enhancement. Lesions in the cerebellum and left pontocerebellar junction.	MS
Sarwar et al. (63)	F47	3 weeks	Fatigue, numbness, blurry vision	N/A	Multiple scattered periventricular lesions with contrast enhancement and hyperintense lesions involving periventricular areas of both hemispheres	MS

A list of reported MS cases following SARS-CoV-2 infection, based on Sarwar 2021. When available, laboratory results for AQP-4, MOG, oligoclonal bands in CSF, and CSF SARS-CoV-2 are listed.

AQP-4, aquaporin-4; CSF, cerebrospinal fluid; F, female; IgG, immunoglobulin G; M, male; MOG, myelin-oligodendrocyte-glycoprotein; MS, multiple sclerosis; MRI, magnetic resonance imaging; N/A, not available; NMO, neuromyelitis optica; ON, optic neuritis; RT-PCR, reverse transcription polymerase chain reaction; SARS-CoV-2, severe acute respiratory syndrome coronavirus 2.

### Neurotropism and Viral Spread in the CNS

**Figure 1:**
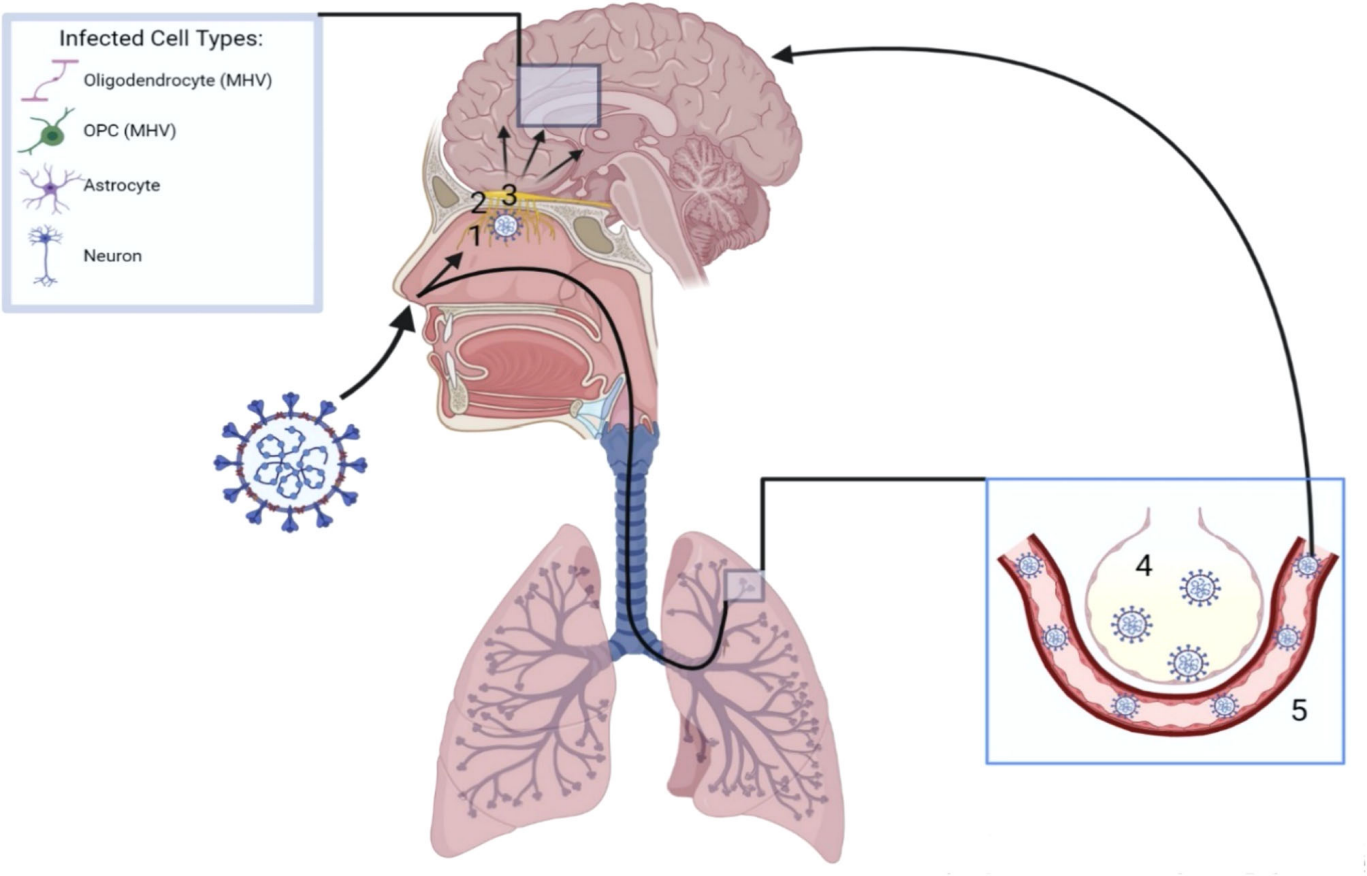
Neurotropism of SARS-CoV-2. Upon inhalation, SARS-CoV-2 can reach the brain parenchyma *via* two mechanisms. Following the olfactory route, SARS-CoV-2 infects the olfactory epithelium (1) directly under the cribiform plate (2). The virus can then traverse the cribiform plate through its foramina or *via* the olfactory nerves, gaining access to the olfactory bulb (3) and spreading to first and second order connections throughout the brain. Additionally, SARS-CoV-2 may be inhaled into the lungs, reach the alveoli (4) and gain access into the blood stream (5). A hematogenous route of SARS-CoV-2 neuroinvasion can then occur following breach of the BBB. Once inside the brain parenchyma, neurons and glial cells such as astrocytes have been shown to be directly infected. Oligodendrocytes and OPCs have shown similar infection with the coronavirus strain MHV.

The high prevalence of anosmia, coupled with the presence of airborne viral particles, suggests an intranasal route of SARS-CoV-2 CNS invasion, particularly through the olfactory pathway (**Figure 1**). Within each nostril, the olfactory epithelium contains bipolar olfactory neurons (72). These neurons extend apically to interact with air particles, as well as basally in small nerve bundles to transverse the cribriform plate of the ethmoid bone, forming olfactory nerves (72). The bony cribriform plate contains foramina which may serve as easy access points for viral brain infection through access to the intracranial space (72). However, viral particles may also spread from the olfactory nerve to the olfactory bulb, following the olfactory tract to brain structures such as the piriform cortex, amygdala, parahippocampal gyrus, olfactory tubercle, and anterior olfactory nucleus (72). Bacterial meningitis can occur through nasal infections (73), and evidence suggests that SARS-CoV-2 follows the olfactory pathway to gain access to the CNS as well. In autopsies of patients that succumbed to COVID-19, high viral presence is detected in the olfactory mucosa underneath the cribriform plate and high levels of SARS-CoV-2 RNA are detected in the olfactory bulb (74, 75). Understanding how SARS-CoV-2 transits from the olfactory bulb to connecting CNS structures is therefore of great interest.

Mouse models of SARS-CoV, SARS-CoV-2, and orthologous murine coronaviruses are useful in studying the spread of viral antigen throughout the CNS. Because SARS-CoV and SARS-CoV-2 do not bind effectively to murine ACE2, transgenic models expressing humanized ACE2 (hACE2) are required to allow the recapitulation of coronavirus infection in mice (76, 77). K18-hACE2 mice, which express hACE2 driven by the cytokeratin 18 (*KRT18*) promoter, allow the expression of hACE2 in lung and brain epithelial cells, as well as in epithelia of the kidney, gastrointestinal tract, and liver (77). These mice show significant viral antigen presence in brain structures following intranasal infection with SARS-CoV, despite low hACE2 expression in the brain compared to the lungs (76). Following intranasal infection, viral antigen presence is observed in the olfactory bulb by 60-66 hours post infection (hpi) (78). The virus then spreads to brain regions with first and second order connections to the olfactory bulb such as areas in the cortex (piriform and infralimbic cortices), basal ganglia, and midbrain (dorsal raphe) (78). This suggests retrograde neuronal migration, which is also seen in rabies and herpes zoster infections (79). Interestingly, intracranial inoculation of even a low dose of SARS-CoV (3.2 PFU) in k18-hACE2 mice causes neuronal inflammation and rapid death by 4 days post infection (dpi), with a high viral presence in the dorsal vagal complex that controls breathing (78). CNS infection may therefore contribute to respiratory issues associated with severe coronavirus-induced pathologies such as SARS or COVID-19.

Murine studies with SARS-CoV-2 show similar neuronal spread, with viral nucleocapsid presence detected in the olfactory bulb and connecting brain regions such as the cerebral cortex, caudate/putamen, thalamus, hypothalamus, and ventral striatum by 6 dpi in k18-hACE2 mice infected intranasally (10^5^ PFU) (80). Thrombi are also observed in the thalamus at this time (80). The involvement of the thalamus is particularly interesting, as greater than half of MS patients present with thalamic lesions (81). Injury of thalamic gray matter in MS has been linked to cognitive and motor impairment, as well as fatigue, pain, and ocular motility issues (81, 82). Viral presence in the thalamus may potentially worsen these symptoms. In addition to showing viral presence in MS associated structures, the mouse model utilized by Zheng et al. (2021) also recapitulated anosmia phenotypes during social scent discrimination and buried food assays, further suggesting the importance of the olfactory pathway in CNS infection (80).

The Perlman group has conducted pioneering work with models of coronavirus infectivity, particularly with the MHV-JHM murine betacoronavirus, which shares 52.5% homology with SARS-CoV-2 (83). Infection with this virus induces CNS demyelination in mice, offering an animal model of MS and a way to study viral spread to CNS white matter. Previous work from the Perlman laboratory also supports the role of the olfactory pathway in facilitating viral spread to the CNS, specifically the olfactory nerve, suggesting this may also be a likely candidate for SARS-CoV-2 neuroinvasion. Unilateral ablation of the olfactory bulb in mice infected with MHV-JHM prevents viral spread on the ablated side (84). In the same mouse model, temporary impairment of the olfactory nerve with the surfactant Triton X-100 prevents CNS viral entry until the reagent wears off and the neuroepithelium begins to regenerate (85).

Tracking viral spread from the brain to the spinal cord offers a way to elucidate virus-induced pathology and disease exacerbation. In fact, extending survivability of MHV-JHM infected mice with low levels of monoclonal antibodies against the virus’ surface (S) glycoprotein leads to infection of the spinoreticular tract at 5 dpi, the timepoint at which spinal cord gray matter shows signs of infection (86). This suggests possible retrograde spread from the brain to the spinal cord (86). Spinal cord white matter is infected in this model by 7 dpi, further showing the potential for coronavirus infiltration in structures associated with MS (86).

A compartmentalized immune response to SARS-CoV-2 could further highlight the differences between direct CNS infection and CNS infection *via* a hematogenous route. To determine whether intrathecal anti-SARS-CoV-2 antibodies are triggered by neuroinvasion and activation by local antigen or by the systemic circulation, an adeno-associated virus (AAV) can be used in mice to express hACE2 in *1)* lungs only, *2)* brain only, or *3)* both lungs and brain (87). Expression of hACE2 permits SARS-CoV-2 infection, since the virus does not bind effectively to murine ACE2 (76, 77). When hACE2 is expressed only in the lungs, intranasal SARS-CoV-2 infection triggers anti-Spike IgG in the lungs and serum of the mice, but not in the brains or CSF (87). When hACE2 is expressed in both lungs and brain, anti-Spike antibodies are identified in all four compartments (87). Lastly, when the receptor is expressed only in the brain and infection occurrs intracranially, anti-Spike antibodies are only detected in the brain and CSF and not the lungs or serum (87). The compartmentalization of the immune response suggests that anti-COVID antibodies do not enter the brain parenchyma from the bloodstream; rather, they are elicited within the CNS (87), presumably by B cells recruited to clear the infection.

### Glial Interactions

**Figure 2:**
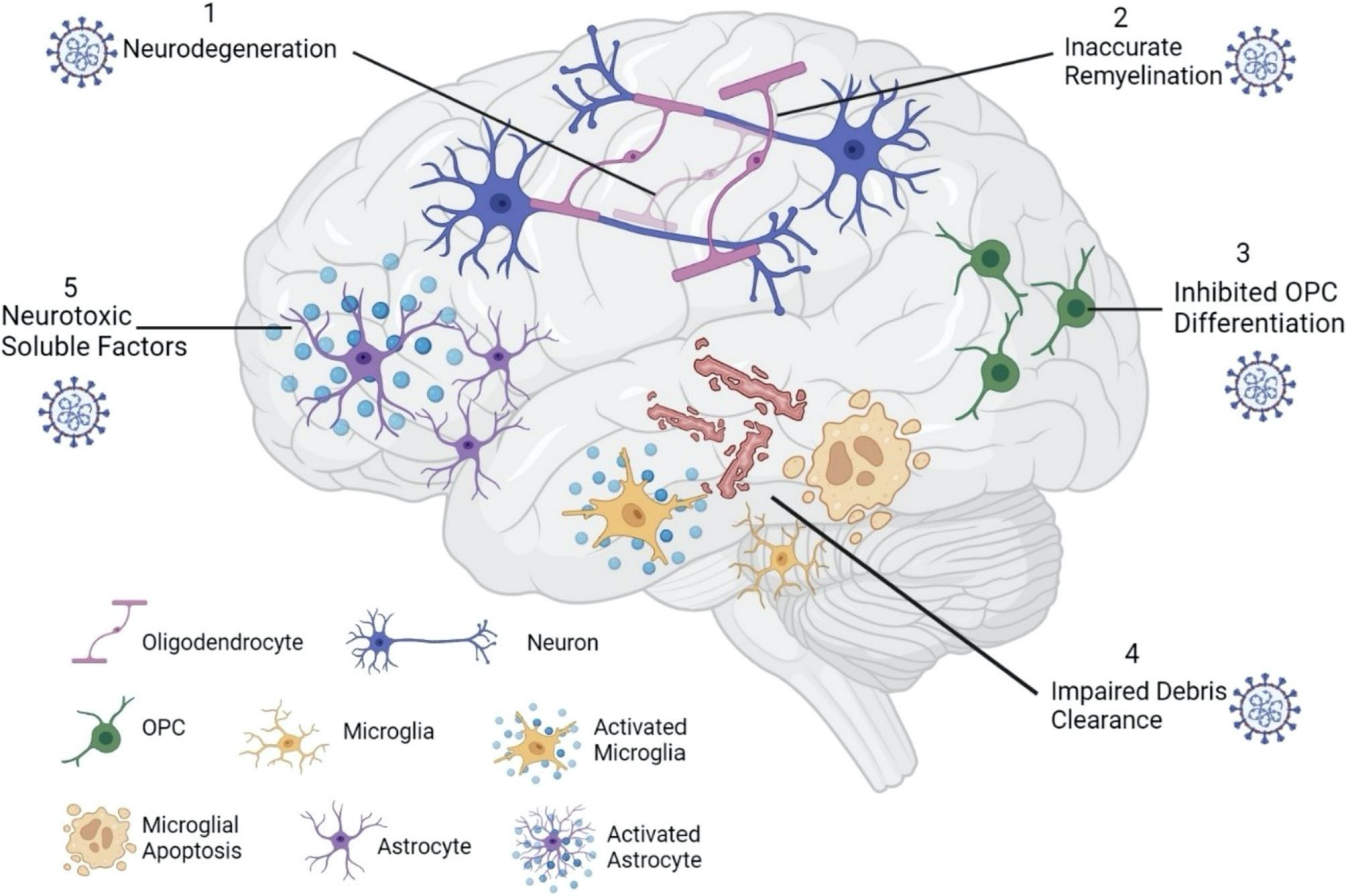
SARS-CoV-2 impacts the glial landscape. Once inside the brain parenchyma, SARS-CoV-2 can alter the neural microenvironment by impacting glial cell function. Infected oligodendrocytes can result in neurodegeneration (1), leading to inaccurate remyelination by surviving oligodendrocytes (2). Inhibited OPC differentiation (3) further impairs the remyelination process, as new oligodendrocytes are not formed. While activated microglia secrete proinflammatory cytokines that are damaging to neural tissue, microglial apoptosis reduces myelin debris clearance (4), further suppressing remyelination potential. Lastly, activated astrocytes may secrete neurotoxic soluble factors (5) that can impair neuronal viability and damage axons.

Once SARS-CoV-2 breaches the CNS, the presence of the virus particles may impact the glial landscape. In the CNS, oligodendrocytes (OLs), astrocytes, and microglia are the major glial types and all three cell types express ACE2 and TMPRSS2 (21, 88, 89). Because glial cells play an integral role in the maintenance of neural microenvironments and secrete inflammatory cytokines in response to injury, examining a potential glial response to SARS-CoV-2 is important in the context of neurodegenerative disorders like MS (**Figure 2**).

#### Oligodendrocytes (OLs)

OLs are responsible for myelin production in the CNS, and oligodendrocyte precursor cells (OPCs) renew the OL pool. Thus, damage to OLs and OPCs in MS induces demyelination while also repressing remyelination (90). Importantly, in recent years it has been shown that OLs and OPCs are not simply passive targets of damage in MS but can also play active roles in MS inflammation and progression, possessing the ability to present antigen and attract CD4^+^ and CD8^+^ T cells that elicit further demyelination (91, 92). Direct coronavirus infection of OLs has been documented previously, as the OL cell line MO3.13 shows acute and persistent infection with human coronavirus 229-E (HuCV-229E) (93).

Because of their direct involvement in MS pathogenesis, the infection of OLs by SARS-CoV-2 could potentially alter, or even worsen, demyelination in MS patients. Indeed, coronavirus-induced demyelination of the brain, spinal cord, and optic nerve has been postulated based on case studies of HCoV-OC43 and SARS-CoV-2 patients (5, 6, 94). Case study reports indicate that symptomatic COVID-19 might even precede demyelinating events. For example, COVID-19 respiratory complications arose two weeks before the onset of demyelination in the cervical and thoracic spine, pons, and corpus collosum in a previously healthy individual (7). Similarly, MS developed *de novo* 3 weeks after a 47-year-old female was admitted to the hospital for COVID-19 (63). Interestingly, in another case, the onset of COVID-19 respiratory issues appeared two weeks before peripheral nervous system demyelination symptoms associated with Guillain Barre syndrome (95). CIS has also been reported in a patient with positive SARS-CoV-2 antibodies in the CSF (96). The fact that some patients present with COVID-19 symptoms before the appearance of demyelinating lesions suggests a potential connection between viral infection and myelin destruction. While anecdotal, these observations suggest that COVID-19 infection may be a trigger for neuro–inflammatory responses.

Mechanistically, it has been documented how coronavirus infection can alter myelination in the CNS. Utilizing the MHV model, Pan et al. (2020) infected flox/STOP TdTomato reporter mice with a Cre-expressing recombinant MHV strain; this results in previously infected and surviving cells permanently expressing the tdTomato protein. Interestingly, tdTomato^+^ cells in this model are mainly identified as OLs, especially in the splenium of the corpus callosum and cervical and thoracic spinal cord (83). At 30 dpi, previously infected TdTomato^+^ OLs in the spinal cord are located in areas with demyelinated lesions and are associated with activated microglia/macrophages, as well as increased levels of CD8^+^ T cells (83). These OLs also show upregulated expression of MHC class I through 90 dpi, as well as upregulated expression of genes involved in antigen presentation (83). As class I-restricted CD8^+^ T cells and their inflammatory responses are strongly linked to MS pathogenesis (97), this data might present a potential mechanism by which coronavirus infection may exacerbate MS.

Additionally, TdTomato^+^ OLs in this model show downregulation of genes required for precursor cell differentiation (83). This is interesting, as TdTomato^+^ cells in the subventricular zone (SVZ) were oligodendrocyte precursor cells (OPCs) rather than OLs, posing the question of whether infection halted or inhibited their differentiation (83). It has recently been shown in a zebrafish model that OLs that survive demyelination are less capable of inducing remyelination, exhibiting less myelin sheath generation and more mistargeted myelin compared to newly generated OLs (98). Coronavirus OL infection may therefore impact the demyelination process in those with MS by increasing the CD8^+^ T cell inflammatory response, as well as delaying or inhibiting remyelination potential by affecting OPCs (83). With OPC differentiation inhibited, the surviving OLs may be forced to attempt remyelination that is mistargeted and inaccurate (98). Thus, both direct damage to OLs, as well as inhibition of OPCs following coronavirus infection has the potential to negatively alter myelination in the CNS.

#### Microglia

While OLs are directly involved in myelin production, microglia serve as the resident immune cells within the CNS, playing roles in debris clearance, synaptic organization, OPC maintenance, and communication between different cell types through the secretion of neurotropic factors (99, 100). In the context of MS, it has been suggested that different subtypes of microglia play distinct roles. Microglia with a proinflammatory phenotype, traditionally classified as M1, tend to worsen demyelination in MS through the production of inflammatory cytokines such as IL-6, IL-8, IL-12, IL-23, IL-1β, and TNF-α (99, 101). They also facilitate antigen presentation and ROS and NO production (99, 101). M2 microglia on the other hand secrete anti-inflammatory cytokines such as IL-4, IL-10, and IL-13, clear myelin debris, and promote the differentiation of OPCs (99, 101). Because of their immune cell function, microglia would likely be implicated in the initial response to SARS-CoV-2 infection of neuronal tissues. Indeed, activated CD68^+^ microglia have been observed in the brains of autopsied COVID-19 patients (102). However, persistent microglial activation can lead to an overproduction of inflammatory cytokines that can damage CNS structures (83). It is thus tempting to speculate that SARS-CoV-2 infection of microglia might shift their phenotype to an inflammatory M1 state, leading to worsening demyelination in MS. One must note, however that the M1/M2 dichotomous paradigm is not entirely accepted, with evidence to support a model in which a continuum of phenotypes exist between M1 and M2 (103, 104).

Multiple lines of evidence indicate that coronavirus infection may trigger microglial inflammation and activation. Treating the mouse microglia BV-2 cell line with the S1 Spike protein of SARS-CoV-2 causes these cells to increase their expression of the inflammatory cytokines TNF-α, IL-6, and IL-1β, as well as augment iNOS, NO, and NF-κB DNA binding and transcriptional activity. The increase in cytokines and reactive oxygen species are hypothesized to result from increased NF-κB signaling (105). *In vivo*, SARS-CoV infection in k18-hACE2 mice also results in microglial activation, although minimal inflammation is observed compared to a JHMV model (78). Further research is needed to address the specific release of cytokines by microglia *in vivo* following SARS-CoV-2 infection, and how this can potentially damage myelin.

In a preprint study, K18-hACE2 mice infected with SARS-CoV-2 also present with microgliosis and microglial activation, as well as a T cell inflammatory response and viral presence in the CNS (106). Despite a lack of CNS demyelination in this model, microglial apoptosis is detected (106). Microglial apoptosis in the context of SARS-CoV-2 is intriguing, as there is evidence that these cells are required for remyelination after coronavirus infection. MHV-infected mice, whose microglia are depleted with the drug PLX5622, fail to recover from paralytic symptoms and show unchanged demyelination levels at 21 dpi, while control mice have reduced demyelination (107). Even at 50 dpi, when control mice are fully recovered from the effects of MHV infection, microglia depleted mice show persistent demyelination and paralysis (107). Higher levels of myelin debris and vacuoles are also observed in these animals, as are reduced OLs in the spinal cord (107). Interestingly, these mice show reduced CD4^+^ and CD8^+^ T cells in the CNS, which normally contribute to demyelination in the MHV model and CNS autoimmune diseases such as MS. This suggests that instead of attenuating demyelination, microglia play a role in remyelination, and that debris clearance by microglia may be necessary for this to happen. This is supported by transcriptional analysis from microglia RNAseq results from microglia of infected mice without PLX5622 treatment, showing upregulation of genes responsible for remyelination, oligodendrocyte maturation, and debris clearance (107). Thus, even if SARS-CoV-2 neuroinvasion does not cause myelin degeneration, the induction of microglial apoptosis (106) might disrupt mechanisms of remyelination in MS-affected individuals.

#### Astrocytes

Astrocytes have additionally been implicated in SARS-CoV-2 infection and COVID-19 and need to be considered in the context of MS exacerbation. In a preprint study, SARS-CoV-2-infected astrocytes have been observed from autopsied COVID-19 patients, and neural stem-cell derived human astrocytes are also shown to be infected following exposure to SARS-CoV-2 for 1 hour *in vitro* (108). This is indicated by the presence of viral genetic information and Spike protein in astrocytes in both autopsy and *in vitro* studies (108). While direct infection of neurons with SARS-CoV-2 does not appear to result in cell death, neuronal viability is reduced when cultured with a conditioned medium of SARS-CoV-2-infected astrocytes (108). Infected glial cells, including astrocytes, may therefore alter the microenvironment by releasing soluble factors that damage surrounding cells. In a preprint manuscript, it has been shown that human cortical tissue cultured with SARS-CoV-2 for 72 hours shows extensive astrocytic infection, as do cortical organoids when infected at peak neurogenesis (22 weeks of differentiation) (109). Increased cellular stress and reactivity are also observed following astrocyte infection, as indicated by increased expression of the endoplasmic reticulum (ER) stress marker ARCN1 and the reactive marker SYNM (109). It is possible that a reactive gliosis state triggered by astrocyte infection could further damage CNS structures.

In patients with severe COVID-19, increased levels of plasma biomarkers for CNS injury, including GFAP (astrocytic activation/injury) and NfL (axonal damage) are exhibited (110). Interestingly, severe COVID-19 patients show a decrease in GFAP levels between initial (a mean of 13 days after onset of symptoms) and follow-up tests (a mean of 11.4 days after initial test), yet an increase in NfL, suggesting initial astrocyte activation followed by delayed neuronal injury (110). Importantly, NfL is a common marker of MS disease. Serum NfL (sNfl) levels are significantly higher in MS patients than in healthy controls and correlate with brain and spinal cord lesions, expanded disability status score (EDSS) assessments, and relapse risk (111). Plasma NfL (pNfL) also appears to correlate with MS severity. For example, a longitudinal study has shown that elevated pNfL in MS-afflicted individuals increases the risk of developing long-term sustained disability (112). As severe COVID-19 appears to trigger axonal damage through astrocyte activation, it is possible that CNS injury in MS-affected individuals may be exacerbated by SARS-CoV-2 infection.

### BBB Disruption

#### General Evidence for BBB Disruption by SARS-CoV-2

Evidence for altered BBB permeability following SARS-CoV-2 infection has been obtained from 2D static and 3D microfluidic *in vitro* BBB models. While SARS-CoV-2 Spike protein does not trigger endothelial cell cytotoxicity *in vitro*, the introduction of the Spike protein subunit 1 (S1), subunit 2 (S2), or receptor binding domain (RBD) results in loss of integrity of a 2D BBB model (113). The model, which consists of a 2D static human brain microvascular endothelial cell (hBMVEC) monolayer, shows significant reductions in electrical resistance, a correlate of BBB tightness, following introduction of Spike protein (113). A 3D microfluidic *in vitro* BBB model, which mimics the three dimensionality of the CNS vasculature, supports these results, as evidenced by increased barrier permeability to a dextran dye following introduction of S1 (113). Discontinuous or absent ZO-1 in this model also suggests tight junction breakdown following SARS-CoV-2 introduction. Human induced pluripotent stem cell (hiPSC)-derived brain capillary endothelial like cells (BCECs) are also shown to be infected with SARS-CoV-2 in another *in vitro* model, accompanied by passage of SARS-CoV-2 RNA through a transwell (114). Taken together, these studies show evidence for potential BBB leakiness following SARS-CoV-2 infection.

Recent animal models also support the idea that the virus impacts the BBB. In C57BL/6 mice, injection of full length S1 Spike protein *via* the tail vein results in a significant decrease in neuronal MFSD2a (115), a protein responsible for BBB structural integrity and omega 3 fatty acid transport (116, 117). Injection of truncated S1, which contains only the RBD, does not decrease MFSD2a in this model, nor does injection of S2, suggesting a role for the N-terminal region of the S1 protein in BBB disruption (115).

In humans, endothelial cells of the BBB expresses ACE2, and it is also upregulated in the vasculature of individuals with dementia and hypertension, two disorders that increase complications due to COVID-19 (113). Brains of autopsied COVID-19 patients show a high presence of viral Spike protein in microvessel endothelial cells, as well as signs of perivascular edema and endothelial cell degeneration (115). Spike protein co-localizes with IL-6 and caspase 3 in the endothelial cells of these individuals, supporting the idea that SARS-CoV-2 infection may induce apoptosis of cellular components of the neurovasculature (115). Interestingly, postmortem studies show that MFSD2a protein is significantly decreased in the brains of COVID-19 infected individuals compared to healthy individuals, similar to results seen in mice (115). Case studies of COVID-19 patients also show high CSF/serum albumin indexes, indicative of BBB disruption (118).

**Figure 3:**
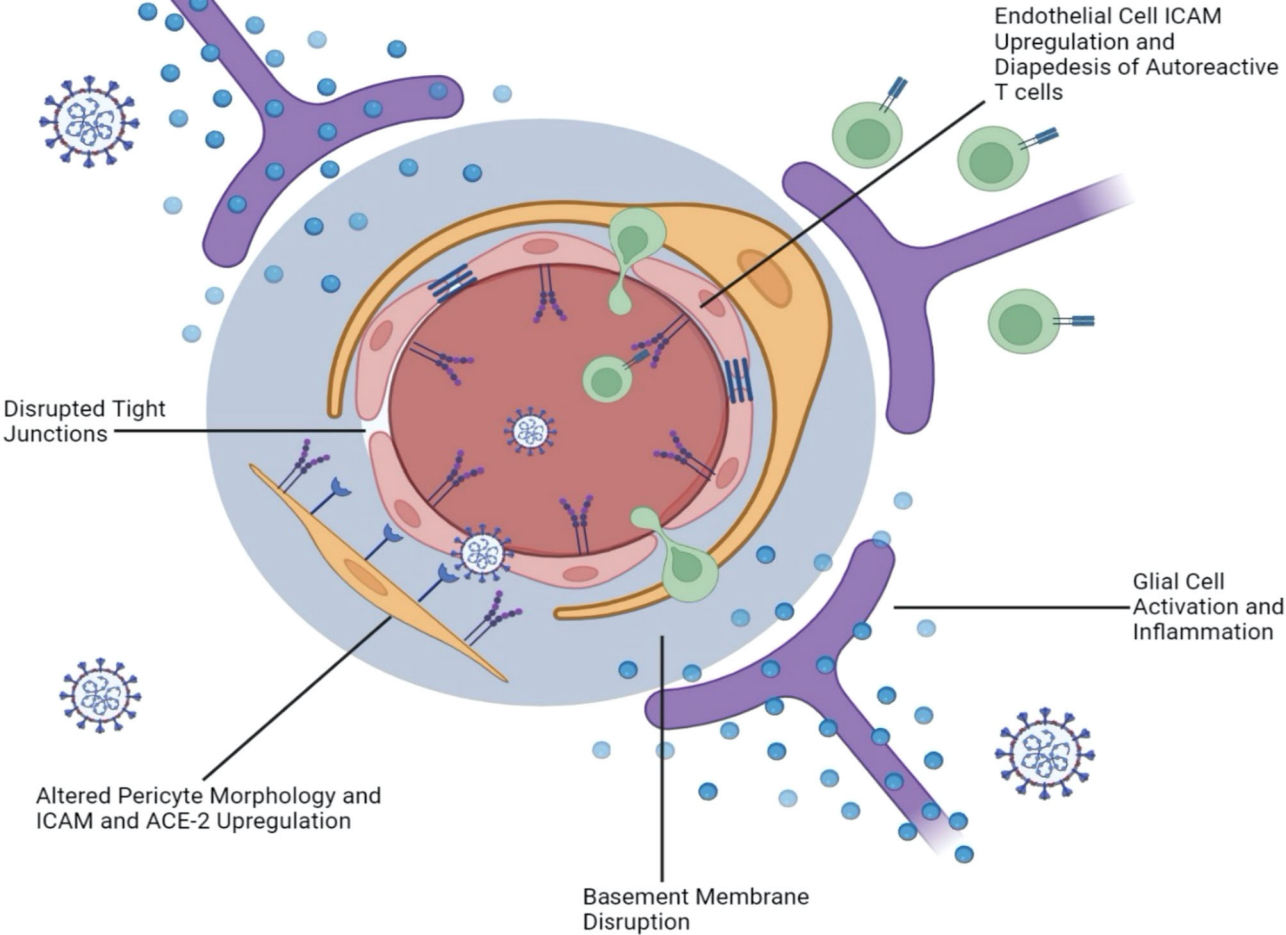
SARS-CoV-2 Damages the Blood Brain Barrier from the “Inside-Out” as well as from the “Outside-In.” Neurotropic infection triggers the activation of glial cells and a proinflammatory response that damages the BBB from within the CNS (inside-out). This is supported by evidence of basement membrane disruption without tight junction alteration, as well as altered cerebral vasculature without direct endothelial cell infection. In contrast, evidence of disrupted or absent tight junctions, as well as upregulated ICAM and VCAM on endothelial cells suggests an outside-in mechanism that allows the diapedesis of autoreactive T cells from the blood, through the BBB, and into the brain. Contracted pericyte morphology coupled with upregulated ICAM and ACE-2 expression also suggests an outside-in mechanism.

With growing evidence that SARS-CoV-2 infection implicates the BBB and the microvasculature, it is important to investigate the mechanisms underpinning this disruption. If SARS-CoV-2 makes its way to the brain parenchyma *via* the olfactory route as we have postulated, it is plausible that a glial inflammatory response to SARS-CoV-2 triggers an “inside-out” weakening of the BBB. It is well documented that cytokines such as IL-6 and IL-17 impact vascular permeability and play a role in MS pathogenesis. As these inflammatory cytokines are released by infected neurons and microglia in the context of both SARS-CoV and SARS-CoV-2 (78, 105), increased permeability of the BBB could lead to further viral neuroinvasion, as well as heightened infiltration of myelin autoantibodies in MS patients. On the other hand, viral particles may also make their way from the peripheral blood to the BBB and trigger leakiness in an “outside-in” route of BBB disruption *via* ACE2, additionally fueling MS exacerbation. In the following sections, we examine the evidence supporting these two pathways to BBB weakening as a result of COVID-19 (**Figure 3**).

#### Evidence for Inside-Out BBB Disruption

*In vivo* models of SARS-CoV-2 infection demonstrate leakiness of the BBB in an “inside-out” fashion. In both k18-hACE2 mice and Syrian hamsters, intraperitoneal injection of Evans blue dye at 6 dpi shows leakage into the cortex when the animals are inoculated with SARS-CoV-2 intranasally (119). Both mouse and hamster brains show elevated levels of inflammatory molecules such as IL-6, TNF-α, and MCP1. Collagen IV, a component of the basement membrane, is decreased in cerebral vessels of the animals, while matrix metalloproteinase-9 (MMP9), which is known to degrade Collagen IV, is increased (119). Interestingly, tight junctions do not appear to be damaged in this model, as there are no significant differences in occludin, ZO-1, or claudin-5 between the infected and mock infected animals (119). Basement membrane disruption in the absence of tight junction alteration thus supports the “inside-out” hypothesis, since the basement membrane is proximal to the CNS parenchyma. In mechanistic *in vitro* BBB co-culture models using k18-hACE2 mice- or hamster-derived BMECs and astrocytes, decreased Collagen IV, increased MMP9, increased inflammatory cytokine mRNA, and unaltered tight junction structures are observed following treatment with SARS-CoV-2. These data further suggest a transcellular pathway of BBB breach, rather than a paracellular breach through tight junctions (119).

Other *in vivo* models suggest that endothelial cells may not be directly infected by SARS-CoV-2, but rather obtain altered orientation or damage from an inside-out infection. For example, one study showed that intranasal inoculation of k18-hACE2 mice with SARS-CoV-2 did not result in infection of endothelial cells in the brain or spinal cord, indicating that these cells were likely not direct targets of the virus (106). However, discontinuous endothelial layers were seen in vessels with leukocyte infiltrate, and both the vessels and infiltrate stained positive for cleaved caspase 3, indicative of apoptosis. Further, SARS-CoV-2 infection in structures with secondary and tertiary connections to the olfactory bulb was consistently observed (106). This may indicate an “inside-out” model of BBB breakdown, as CNS infection may be required to trigger alterations in the vasculature.

Further evidence supporting the idea of SARS-CoV-2-induced damage to the neurovascular network comes from Song et al. (2021), who found no viral presence in the vascular endothelium of intranasally-infected k18-hACE2 mice but did observe disruption in the cortical vasculature at 7 dpi. After labelling for nucleocapsid protein, CD31, and Podocalyxin, a whole brain vascular reconstruction was made using ClearMap (120). Neural cells with high viral expression coincided with abnormal density and orientation of the vascular network (120). As the vascular endothelium was negative for SARS-CoV-2 expression, these results suggest that CNS infection can lead to vasculature alteration and potential “inside-out” damage to the BBB (120).

In summary, even if SARS-CoV-2 infection is not, on its own, sufficient to trigger MS-like demyelination in infected subjects, the likelihood of an altered or disrupted BBB appears high. In individuals with existing MS, further weakening of an already compromised BBB from the inside-out by COVID-19 could further damage oligodendrocytes and exacerbate existing demyelination, thus contributing to exacerbation of symptoms.

#### Hypoxia: A Potential Factor in Demyelination?

With growing evidence that SARS-CoV-2 can alter the neurovasculature, the potential for microthrombi in the large vessels of the brain should be addressed, as this can lead to reduced blood flow and ischemia. Large vessel occlusion is frequently observed in around half of COVID-19 patients, as well as ischemic stroke (121). Microthrombi and acute infarction are seen in the brains of autopsied COVID-19 patients (74, 122, 123), accompanied by elevated D-dimer and fibrinogen (122, 124). Ischemic infarcts in subcortical white matter have been found following brain autopsies, as well as tissue damage and cell death (120). In a case series of 50 patients assessed a minimum of 6 weeks following a confirmed SARS-CoV-2 infection, Fogarty and colleagues found that, compared to controls, these patients had pro-thrombotic changes and elevated EC activation markers (125). Together, these findings are suggestive of an underlying endotheliopathy and could explain the high prevalence of thrombotic events in convalescent COVID-19 patients (126).

Further, studies utilizing human brain organoids have demonstrated how virally induced hypoxia can damage nearby cells. When incubated with SARS-CoV-2, infection is seen in neurons, radial glia, and neuronal progenitor cells, but only 15% of infected cells show evidence of cell death (120). Rather, infected cells appear to promote death of nearby cells, possibly *via* hypoxia, as evidenced by increased HIF-1α staining (120). This, coupled with hypoxia triggered from altered neurovasculature and ischemia, could play a role in oligodendrocyte damage and interfere with myelination.

An “inside-out” mode of altered vasculature and subsequent hypoxia has the potential to severely damage the neural landscape in those with MS, particularly oligodendrocytes. A study utilizing zebrafish to investigate the effect of hypoxia on myelination showed that hypoxia is linked to suppressed OPC migration, decreased oligodendrocyte myelination, and reduced myelin basic protein (MBP) (127). Hypoxia has also been linked to reduced myelinogenesis and motor impairment in adult mice, likely from inhibiting OPC differentiation (128). This raises the possibility that SARS-CoV-2 triggered hypoxia may exacerbate CNS myelin damage in MS patients, as reparative and remyelination mechanisms appear to be suppressed in hypoxic states. Indeed, hypoxia has been observed in both mouse and human demyelinating lesions, suggesting it plays a role in MS. For example, in an LPS triggered EAE model of MS, hypoxia is evident in the gray and white matter of rat spinal cords prior to the onset of demyelination (129). Interestingly, autopsy material from patients with MS, patients with virally induced (HSV, CMV, and PML) inflammatory white matter lesions, and patients with acute ischemic stroke, show similar trends of decreased myelin associated glycoprotein (MAG), oligodendrocyte apoptosis, and significant nuclear HIF-1α expression (130). SARS-CoV-2 induced hypoxia *via* vasculature alteration or other mechanisms may therefore increase the demyelinating process in MS afflicted individuals.

#### Evidence for “Outside-In” Neuroinvasion

Some viruses such as West Nile Virus, HIV-1, and Zika Virus, gain access to the CNS *via* the hematogenous route, by either infecting circulating cells capable of passing the BBB, or by directly altering the integrity of the BBB and its endothelial cells (131–134). Here, we examine evidence that SARS-CoV-2 may also use a hematogenous route to invade the CNS from the outside-in.

Following infection of hiPS-BCECs from the apical side of a transwell dish (representing the vessel), SARS-CoV-2 N and Spike proteins are detected in a dose dependent manner in these cells starting at 1 dpi (114). A significant increase in SARS-CoV-2 RNA is seen on the basolateral side (representing the brain parenchyma) as early as 16 hpi, although permeability to fluorescein protein is unchanged (114). Thus, tight junctions may not be directly disrupted by SARS-CoV-2; however, the virus may breach the BBB *via* transcellular passage in vesicles, mimicking “outside-in” infiltration (114). *In vivo*, injection of radiolabeled S1 protein (I-S1) revealed that it readily crosses the BBB in comparison to radiolabeled albumin, which crosses poorly (135). This was shown to occur *via* adsorptive transcytosis, a mechanism in which proteins bind to endothelial glycoproteins and travel across the cell membrane *via* vesicles (135). Interestingly, intravenous injection resulted in ten times more whole-brain I-S1 than intranasal injection in this model, and low amounts of I-S1 could be detected in the blood following intranasal injection (135). This suggests that the hematogenous route of SARS-CoV-2 infection may in fact be more efficient than the “inside-out” route (**Figure 1**).

ACE2 is expressed in endothelial cells of the BBB, pericytes, and astrocyte endfoot processes, which extend to the blood vessels (136). ACE2 expression is heterogenous in pericytes, with some patients exhibiting moderate to high levels of ACE2, and others expressing close to none (137). Of note, an analysis of brains of COVID-19 patients at autopsy indicated that neurological symptoms were only present in those with moderate-to-high levels of perivascular ACE2 expression and subsequent BBB leakiness (137). In human brain vascular pericytes, ACE2 expression is significantly upregulated in a dose dependent manner following *in vitro* stimulation with SARS-CoV-2 Spike protein, as well as in pericytes exposed to Spike protein and hypoxia (138). A contracted and elongated morphology accompanied by an increase in ICAM, IL-18, and MIF expression is also seen in these pericytes, which is enhanced by hypoxia (138). In k18-ACE2 mice infected intranasally with SARS-CoV-2, ACE2 expression in pericytes is also increased, and pericytes exhibit a reactivity pattern similar to that seen after ischemic stroke (138). An upregulation of ACE2 may facilitate the neuroinvasion of SARS-CoV-2 through the BBB and into the brain parenchyma, while an altered pericyte morphology may be harmful to the BBB structural integrity. An increase in ICAM and inflammatory pathways could also increase the diapedesis of immune cells in an “outside-in” manner, further allowing BBB breach and potential damage to glial cells such as oligodendrocytes.

The pathophysiology of long COVID may feature a sustained increase in BBB permeability, with evidence suggesting that endothelial cell activation may play an important role. Chioh and colleagues found elevated levels of circulating endothelial cells (CEC), a marker of vascular injury, in post-COVID-19 patients (139). The CEC elevation in these patients correlated to elevated pro-inflammatory cytokine levels, such as IL-8, IL-17A and IL-18, suggesting persistent immune activation as a trigger of EC activation. Evidence of direct endothelial cell infection is of importance when evaluating possible entry routes of SARS-CoV-2 in the CNS. It could suggest that upregulation of ACE2 in the brain vasculature could permit SARS-CoV-2 virus to gain access to the CNS directly after infecting endothelial cells. Notably, hBMVECs incubated with all three Spike protein subunits also trigger an increase in ICAM-1 and VCAM-1 after 4 hours, which remain elevated by 24 hours. ICAM-1 and VCAM-1 play essential roles in T cell extravasation from the blood into tissues, including the diapedesis of particles across the BBB into the brain parenchyma (140). Virally induced inflammation coupled with upregulated ICAM-1 and VCAM-1 could allow SARS-CoV-2 to breach the BBB in an “outside-in” manner. Further, elevated levels of the leukocyte chemotaxis factors CXCL10 and CCL5, the pro-inflammatory cytokines IL-1β and IL-6, and numerous MMPs are also observed following exposure to each of the subunits (113). In the context of MS, elevated ICAM-1, VCAM-1, and leukocyte chemotaxis factors may not only increase the potential for SARS-CoV-2 neuroinvasion but may also increase the diapedesis of autoreactive T cells into the CNS where they can attack and destroy myelin. Further degradation of the BBB by inflammatory cytokines and MMPs have the potential to facilitate this process. It is thus important to consider the hematogeneous route of SARS-CoV2 neuroinvasion, as the BBB can be attacked from both the “inside-out”, as well as the “outside-in”.

#### Evidence for “Outside-In” via the Choroid Plexus

Studies also suggest a role for the choroid plexus in SARS-CoV-2 neurotropism. Transcriptome studies of the choroid plexus from COVID-19 patients show that the structure expresses genes for SARS-CoV-2 entry and has upregulated inflammatory genes, as well as upregulated *IFITIM3*, which is a gene responsible for antiviral defense (102). Choroid plexus inflammation is also observed in the brains of postmortem COVID-19 patients (120). Infection of the choroid plexus presents an alternate route for viral and leukocytic entry into the CNS, likely in an “outside-in” manner from the blood into the CSF and brain parenchyma.

SARS-CoV-2 infection has been observed in choroid plexus organoids (141, 142), and viral breach may be due to loss of integrity of the barrier following infection. Genes involved in cell junction structure are downregulated in infected organoids (141), and disrupted tight junctions are also observed, as evidenced by irregular claudin-5 staining (142). An upregulation of inflammatory cytokines such as CCL7, IL-32, CCL2, IL-18, and IL-8 (3) may further allow the weakening of the choroid plexus and permit the entry of SARS-CoV-2 and leukocytes into the CNS, resulting in further neuroinflammation and damage to CNS structures such as myelin.

### Molecular Mimicry and Generation of Autoantibodies

**Figure 4:**
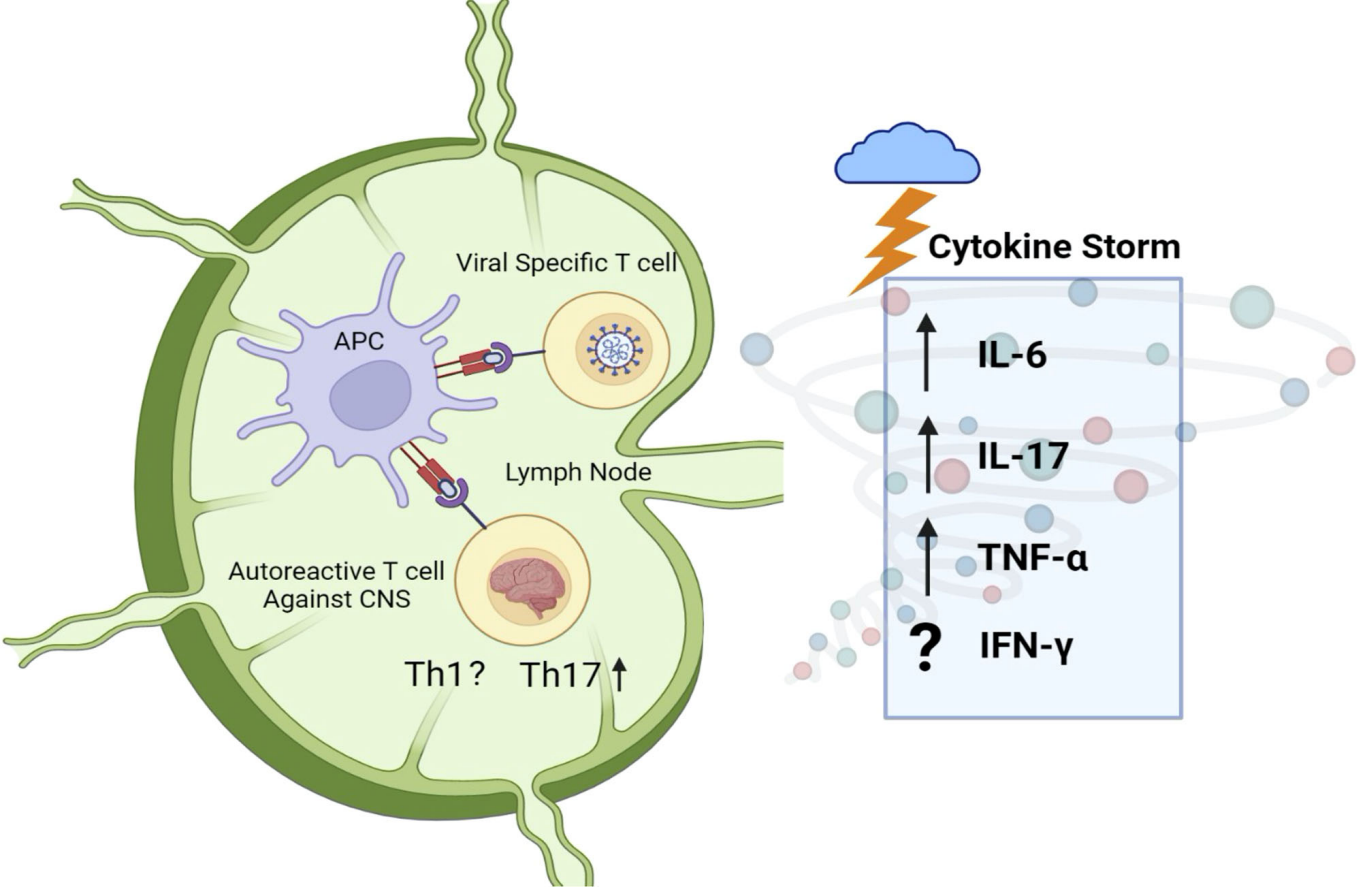
Peripheral immune activation and molecular mimicry against myelin. The adaptive immune response to SARS-CoV-2 may result in the activation of CNS-autoreactive T cells as a result of molecular mimicry. Cytokine storm, observed in SARS-CoV-2 infected individuals, could then elicit the differentiation of CNS-antigen-specific Th17 cells. The upregulation of IL-6, IL-17, and TNF-α is of particular interest, as these cytokines are heavily linked to MS and other demyelinating disorders in mouse and man such as NMOSD and EAE. Further research is needed to understand the Th1 response and generation of IFN-γ in response to COVID-19, as literature suggests mixed results.

Relapse-onset MS is well established as a T cell autoimmune disease. The possibility of cross-reactivity of SARS-CoV-2-specific T cells with myelin antigen (molecular mimicry) is thus an attractive one. T cell clones generated from blood samples of MS patients have previously shown to be cross-reactive for human coronaviruses (HCoV-229E and HCoV-OC43) and myelin, with HCoV-229E and MBP appearing to be the two antigens most involved in cross-reactivity (143). An activated coronavirus-specific T cell clone in the immune periphery could conceivably infiltrate the CNS and elicit or worsen tissue damage. Indeed, cross-reactivity to both myelin basic protein and coronavirus epitopes is observed in T cell clones from MS-affected individuals (**Figure 4**).

Recent literature suggests that SARS-CoV-2 can in fact trigger the production of autoantigens against neural structures. In a study of eleven SARS-CoV-2 positive patients in Germany, two patients were identified to have myelin autoantibodies in their serum and CSF (144). The CSF was analyzed with indirect immunofluorescence on unfixed mouse brains to identify autoantibodies not detected by normal assays (144). This demonstrated goat anti-human IgG staining on numerous neuronal structures such as the hippocampus, olfactory bulb, vessel endothelium, astrocytic proteins, neuropil of the basal ganglia, glial limitans, and myelinated fibers in the cerebellum (144). The specific observation of autoantibodies against myelin and vascular components is interesting, as one could imagine that damage to myelinated structures and the BBB would be harmful to COVID-19 patients with MS. While the study size was small, the fact that autoantibodies for neural structures were detected highlights the potential for virally induced molecular mimicry.

In a preprint study, 408 proteins with high dermatan sulfate (DS) affinity have been uncovered; DS often binds released autoantigens (145). Using bioinformatics, this “autoantigenome” was compared to proteins that were altered at the protein and RNA levels in COVID-19 patients and SARS-CoV-2 infected cells. This analysis identified numerous autoantigens that were highly associated with the nervous system, including 23 autoantigens for the olfactory bulb and 26 for the myelin sheath (145). This is intriguing, as it could explain case studies showing demyelination following SARS-CoV-2 infection (7, 95). As an example, a recent case study of a SARS-CoV-2 positive 26-year old man presenting with optic disc edema, bilateral optic neuritis, and short lesion in the cervical spine, showed the presence of MOG-IgG antibodies (51). The patient reported experiencing a progressive dry cough a few days before the occurrence of vision issues and lacked a personal or family history of demyelination or autoimmunity (51). The MOG autoantibodies and subsequent CNS demyelination were thus thought to be triggered by SARS-CoV-2 infection (51). Demyelination following viral infection is not unheard of, as it also occurs in acute disseminated encephalomyelitis (ADEM), a phenotype of MOG-associated disease (146).

Neural autoantigens were detected in another study in which molecular mimicry was suspected (87). The CSF and blood of COVID-19 patients with neurological symptoms were analyzed to determine if compartmentalized immune responses occur in response to the virus (87). Numerous antineural autoantibodies were discovered in the CSF through anatomic mouse brain immunostaining, including that for cortical neurons, the olfactory bulb, thalamus, hippocampus, cerebellum, brain stem, and cerebral vasculature (87). As the thalamus is another important relay and plays a significant role in MS pathology, and antibodies targeting most of these structures were also demonstrated in Franke et al. (144), these data further highlight the potential for SARS-CoV-2 to trigger autoimmune responses that could potentially exacerbate MS symptoms.

### Cytokine Storm and Peripheral T Cells

COVID-19 pathogenesis involves an initial viral replication phase, responsible for the flu like symptoms and pneumonia, that can progress in some cases to a multi-systemic inflammatory disease, with an exaggerated immune response characterized by cytokine storm (147). Acute respiratory distress syndrome (ARDS) is one example of a secondary organ dysfunction that was observed in up to 20% of COVID-19 cases early in the pandemic (148). The biphasic disease model is supported by the RECOVERY trial (149) in which dexamethasone, an immunosuppressive drug, showed great benefits in severely ill patients (severe and critical disease), but did not improve outcomes in patients that did not need supplemental oxygen (mild to moderate disease). The inflammatory response in COVID-19 patients is mediated by cytokine storm, that may be induced by pro-inflammatory T cells. Indeed, persistent activation of CD4^+^ and CD8^+^ T cells in COVID-19 patients is associated with severe disease and worse outcomes (150).

Fajgenbaum and June have proposed a unifying definition of cytokine storm based on three criteria: *1)* elevated circulating cytokine levels, *2)* acute systemic inflammatory symptoms and 3) excessive secondary organ dysfunction due to inflammation (if a pathogen is present), or any cytokine-driven organ dysfunction (in the absence of pathogen) (55). Numerous cytokines have been identified as being increased in the circulation of COVID-19 patients, such as IL-6 (14, 68, 151, 152), as well as IFN-γ and IL-17 (69). Other elevated cytokines in COVID-19 patients include TNF-α, G-CSF, IL-2, and IL-7, as well as the anti-inflammatory cytokines IL-4 and IL-10 (11, 151, 152). Here, we focus on IL-6, IL-17, and IFN-γ, given their known involvement in MS and other neuroimmunological disorders (**Figure 4**).

#### IL-6

IL-6 is a prominent pro-inflammatory cytokine in the innate immune response to pathogen, often released by macrophages, endothelial, epithelial, and mast cells (153). A significant increase of IL-6 in the blood can lead to cytokine storm, as it triggers immune cell recruitment to the infection site, contributes to endothelial cell damage and vascular permeability, and increases the likelihood of ARDS, multi-organ failure, and death in cases with systemic inflammation (153). Coronavirus infections can trigger the release of cytokines such as IL-6 from monocytes, macrophages, and dendritic cells, leading to either the classic *cis* or *trans* signaling pathways (148). While the *cis* pathway can result in Th17 cell differentiation, an increase in CD8+ T cells, and a decrease in Treg cells, the *trans* pathway can trigger an increased proinflammatory response that can damage vasculature (148). In human airway epithelial cells, the nucleocapsid protein of SARS-CoV appears to stimulate IL-6 expression (154). In the murine nervous system, SARS-CoV infection triggers IL-6 expression by neurons and astrocytes (78), while SARS-CoV-2 triggers IL-6 expression by microglia *in vitro* (105), suggesting a potential role for IL-6 in neurological damage seen by coronaviruses. IL-6 expression is also seen in the brains of SARS-CoV-2 infected mice and hamsters, as well as from infected BMECs from these animals, further supporting IL-6’s role in SARS-CoV-2-triggered vasculature damage (119).

Given its significant upregulation and role in SARS-CoV-2-triggered cytokine storm, it is unsurprising that elevated IL-6 is associated with poor prognosis (14, 68, 151, 152). Specifically, IL-6 serum levels are higher amongst patients with moderate to severe COVID-19 (151). Additionally, a meta-analysis revealed that serum IL-6 concentration was, on average, 2.9-fold higher in complicated COVID-19, defined as the presence of ARDS, ICU admission, or severe/critical COVID-19, as compared to their non-complicated counterparts (68). Heightened IL-6 is associated with respiratory failure and the need for mechanical ventilation, marking it as an important cytokine in COVID-19 pathogenesis (155).

Researchers have suggested the use of IL-6 blocking agents such as tocilizumab and sarilumab for therapeutic benefit, but clinical trials have produced varied results. One clinical trial showed no significant benefit of tocilizumab treatment in preventing intubation or death in COVID-19 patients (46), while others have shown significant reductions in mechanical ventilation and death, as well as significant improvements in clinical outcomes in patients treated with tocilizumab or sarilumab (156, 157). Metanalyses tend to favor the use of IL-6 blocking agents in improving patient outcomes (158, 159). One has even showed that IL-6 concentrations in the blood before drug administration can predict the outcome of tocilizumab treatment, with initially high IL-6 correlated to improved treatment success compared to initially low IL-6 (160).

Upregulated IL-6 in COVID-19 patients may lead to MS exacerbation, as this cytokine has consistently been linked to worsened demyelination and histopathological scores in EAE models. IL-6 deficient mice appear resistant to EAE, as evidenced by significantly reduced clinical scores, demyelination, and inflammation in the CNS (161, 162). The activation and differentiation of MOG-specific T cells is dampened in IL-6 deficient mice, suggesting this process is dependent on IL-6 (162). Anti-IL-6R monoclonal antibodies (anti-IL-6R-mAbs) have been shown to significantly reduce the onset and severity of EAE induced inflammatory infiltrates and demyelination in mice (163). An increase in IL-6 due to SARS-CoV-2 may therefore lead to worsened MS symptoms, specifically demyelination. The fact that SARS-CoV infection triggers upregulated IL-6 in astrocytes is also intriguing (78), as astrocytic IL-6 has been shown to play a significant role in EAE development (164). Specifically, astrocyte-IL-6-KO mice (Ast-IL-6-KO) show delayed onset of EAE pathology and significantly less inflammatory infiltrates and demyelination in the spinal cord (164). While this effect is only observed in female Ast-IL-6-KO mice (164), the evident role of IL-6 in demyelination supports the potential for an IL-6 driven exacerbation of inflammation and demyelination in SARS-CoV-2 positive MS patients.

IL-6 also plays a significant role in the pathogenesis of NMO and NMOSD. Individuals with NMO that are seropositive for anti-AQP4 show significantly higher CSF concentrations of IL-6 than patients that are seronegative (165). IL-6 concentrations in the CSF are also positively correlated with the length of spinal cord lesions in these patients (165). Additionally, risk of relapse is correlated with IL-6 plasma concentration and with monocyte production of IL-6 (166). IL-6 may additionally play a role in reducing BBB integrity in NMOSD (167). Interestingly, tocilizumab and satralizumab show promise in treating NMOSD, as they appear to significantly reduce relapse rate in patients, further implicating the role of IL-6 in this demyelinating disorder (168).

The fact that IL-6 is linked to both EAE and NMOSD shows its importance in demyelinating events and suggests a negative effect of heightened IL-6 in SARS-CoV-2 positive individuals with MS. Notably, IL-6 plays a critical role in the differentiation of Th17 cells, acting in concert with TGF-β (169) to drive this subset at the expense of Treg (170, 171). As IL-17^+^ Th17 cells and IL-17 play critical roles in MS pathogenesis, their response to COVID-19 is also of interest (36, 54).

#### IL-17 and Th17 Cells

Recent data has implicated the role of IL-17 in COVID-19 pathogenesis. Transcriptomics analyses showed upregulated IL-17 signaling in various cell types infected with SARS-CoV-2, including those of the lung, heart, and liver (172). IL-17 upregulation appears stronger in SARS-CoV-2 relative to SARS-CoV or MERS-CoV, suggesting a robust IL-17 response that is unique to SARS-CoV-2 (172). IL-17 production is also increased upon COVID-19 infection in humans. While some have suggested IL-17 is significantly elevated in mild COVID-19 patients compared to those that are not infected (69), other studies have shown upregulated IL-17A in COVID-19 patients regardless of disease severity (173).

COVID-19 pathology may also shift the relative balance of Th17 versus Treg. In a study of 40 COVID-19 patients admitted to the ICU, Th17 cell frequency was upregulated in peripheral blood compared to healthy controls (174). The increase in Th17 cells was accompanied by a significant increase in the Th17 transcription factor RORγt, as well as an increase in IL-17 and IL-23, indicating a significant Th17 response (174). Interestingly, a significant reduction in Treg cells was seen in COVID-19 patients compared to controls, accompanied by a reduction in the Treg transcription factor FoxP3 and a decrease in Treg related cytokines such as IL-10 and TGF-β (174). The same trends were observed in COVID-19 patients that succumbed to the disease compared to recovered patients, indicating a substantial role of Th17 driven IL-17 response in COVID-19 severity (174). Neutrophils may also play a role in the promotion of Th17 induction upon SARS-CoV-2 infection, as co-cultures of SARS-CoV-2 infected neutrophils and T cells appear to shift the resulting response towards Th17 and away from IFN-γ^+^ Th1 (175).

An increase in mortality of patients from IL-17 upregulation seems plausible, as upregulated IL-17A has been associated with acute respiratory distress syndrome (ARDS) and diseases that increase COVID-19 complications such as hypertension, diabetes, and obesity (176, 177). Indeed, IL-17 is elevated in the peripheral blood of ARDS patients, as well as the bronchoalveolar lavage (BAL) fluid in mice with LPS-induced acute lung injury (ALI) (178). IL-17 is correlated with ALI in these mice, and IL-17 deficient mice show significantly less severe ALI, as do mice treated with neutralizing antibodies against IL-17 (178).

Accessory proteins such as the open reading frame 8 (ORF8) may be responsible for an increase in IL-17 driven inflammation in COVID-19 patients, as it has been shown to interact with the IL-17A receptor (IL17RA) and stimulate the IL-17 pathway, even in IL-17 deficient cells (179). Mice treated with IL17RA antibodies are protected from IL-17 pathway activation (179). Interestingly, ORF8 has also been linked to the inhibition of the type I interferon (IFN-I) response (180, 181). It has been suggested that COVID-19 severity is driven by dampened antiviral IFN-I and IFN-III responses, coupled with an excess of proinflammatory cytokines such as IL-6 and TNF-α (182–184). An activated IL-17 inflammatory pathway, a weakened antiviral response, and an abundance of proinflammatory cytokines triggered by ORF8 is therefore likely to trigger a cytokine storm that could lead to an excessive inflammatory microenvironment.

The growing literature surrounding the role of IL-17 in COVID-19 has led some researchers to propose inhibiting Th17 and IL-17 for therapeutic benefit (177, 185). The IL-17 antagonist netakimab has shown promising results (186, 187). In a retrospective study of COVID-19 patients treated with netakimab, the IL-6 antagonist tocilizumab, or no treatment, the netakimab treated group showed significantly less mortality, ICU admission, and mechanical ventilation requirement than the other two groups (187). Individuals in the netakimab group also showed significant reductions in lung lesions and no longer required oxygen support (187). In another study, COVID-19 patients taking netakimab showed significant improvement in parameters such as respiratory rate, body temperature, and spO2/FiO2, but clinical outcomes such as mortality rates were not impacted (186). A clinical trial for the IL-17A monoclonal antibody Ixekizumab is also underway (188).

Increased serum levels of IL-17 could plausibly lead to MS exacerbation, as it has been closely linked to its pathogenesis. Th17 cells are involved in the recruitment of neutrophils *via* IL-17, IL-21 and IL-22 release (54). They have been linked to the pathogenesis of several autoimmune disorders, including MS, and contribute to BBB disruption, microglial activation, and astrocyte dysregulation in EAE models (53). During MS relapses, increased frequencies of IL-17-producing CD4^+^ T cells have been detected in both peripheral blood and CSF (36). Th17 cells are also involved in the activation of CD8^+^ T cells. Further, they can directly damage oligodendrocytes (52) and may also be involved in the axonal damage seen in MS (36). Indeed, it is hypothesized that Th17, *via* IL-17, inhibits the maturation and survival of oligodendrocytes (53). In RRMS, elevated levels of IL-17A correlate with an increased CSF to serum albumin quotient (Qalb), suggesting a role of IL-17 in BBB disruption and loss of integrity (189). High levels of IL-17 and Th17 have been documented during MS relapses, both in the periphery and in the CSF (36). Th17 is now considered, along with Th1, one of the main players in MS relapses.

#### IFN-γ

Th1 cells are characterized by their release of the pro-inflammatory cytokine IFN-γ, and both recruit and activate macrophages (190). They have historically been considered key drivers in MS, having been linked to disease onset and progression (54), and to inflammatory activation within the CNS (53). Given that it is a noted player in cytokine storm (55), one might expect that IFN-γ production and, by extension, Th1 activation, may characterize COVID-19 pathology.

Surprisingly, however, the evidence is somewhat mixed. On the one hand, in a study of COVID-19 patients with ARDS, SARS-CoV-2-specific T cells robustly secreted Th1-related cytokines such as IFN-γ, TNF-α, and IL-2 (191). Further, IFN-γ was found to be amongst the most upregulated cytokines in COVID-19 patients, along with IL-6 and TNF-α; concomitant blockade of IFN-γ and TNF-α rescued mortality in k18-hACE2-Tg mice that were infected with SARS-CoV-2 (192).

On the other hand, other studies have drawn the conclusion that IFN-γ production and Th1 activity may actually be diminished in the context of COVID-19 pathology. Advanced age and comorbidity index are associated with lower overall levels of IFN-γ secreting T cells in COVID-19 patients, and patients who recover from a mild disease also appear to have a higher expression of IFN-γ T cells compared to ICU patients (193). Furthermore, severe COVID patients have lower levels of circulating IFN-γ as well as reduced frequencies of CD4^+^ and CD8^+^ T cells as compared to those with moderately severe pathology (151), and polyfunctional Th1 cells are present in lower frequencies in patients with COVID-19. These data hint at a failure to generate a robust Th1 response to SARS-CoV-2 (194). It is important to keep in mind that Th1 cells are considered protective in the context of viral infection – therefore, impaired Th1 responses to acute infection might allow SARS-CoV-2 infection to persist, ultimately resulting in more severe pathology. Further, IFN-γ production is not restricted to T cells; it is a key effector cytokine produced by macrophages and NK cells, and thus impaired Th1 functionality might not present the full picture with respect to the role played by IFN-γ in COVID-19 disease. Further work is needed to clarify a potential connection between COVID-19-induced cytokine storm and IFN-γ/Th1-mediated exacerbation of neuroinflammation.

## Conclusion

MS pathophysiology is multi-factorial, with strong evidence pointing toward a potential viral trigger, such as EBV. The goal of this hypothesis paper was to present the evidence supporting that SARS-CoV-2 may also act as a viral trigger for MS and other neuro-inflammatory diseases, by altering the CNS cellular micro-environment by two potential routes of entry. We propose a model in which SARS-CoV-2 infection and subsequent COVID-19 pathology might exacerbate MS or related disorders such as NMOSD. SARS-CoV-2 can directly infect the CNS *via* the olfactory bulb and the cribriform plate, resulting in neuronal damage and the inhibition of OL differentiation and remyelination. These pathologic responses are augmented by microglial cell death, leading to impaired clearance of myelin debris, and by the secretion of neurotoxic soluble factors from activated astrocytes. In parallel, SARS-CoV-2 can elicit the generation of CNS-specific lymphocytes *via* molecular mimicry, while favoring the differentiation of key Th subsets pertinent in MS, *via* the elicitation of cytokine storm. Crucially, the neurotropic and hematogenous routes of infection and pathology converge on the BBB, which is weakened from both the inside and outside, thus facilitating the infiltration of peripheral immune cells into the CNS parenchyma.

The potential effect of SARS-CoV-2 infection on MS is two-fold. First, it may trigger MS relapses in affected individuals as an immediate consequence to a pro-inflammatory reaction. Second, it can alter more permanently the CNS structural and cellular environment in infected individuals, increasing the risk of developing MS in the long-term. We note that these two mechanisms are currently speculative, at this point, as published data on a direct link between MS and SARS-CoV-2 in humans is scarce. A number of case studies have reported neuroimmunological events, occurring shortly after SARS-CoV-2 infection, as expected in secondary auto-immune events. However, caution should be used in interpreting case reports, given the sheer number of SARS-CoV-2 infections worldwide and the possibility of reporting bias; one cannot infer whether these cases happened by chance or not, in people who happened to be recovering from COVID-19. Few retrospective studies have been conducted so far to examine exacerbation of MS symptoms following SARS-CoV2 infection, and those identified show mixed results. Large-scale epidemiological studies would be needed, to show an increase in incidence of the disease or in its relapse rate during COVID-19 waves or in the years to follow. MS is a chronic disease and its diagnosis can be made years after an initial pathological event. This is a limitation in establishing a link between SARS-CoV-2 infection and MS. The use of disease modifying therapies in MS and the widespread COVID-19 vaccination campaign can also alter a potential causal link between SARS-CoV-2 and MS, respectively by affecting immune cells and their cytokine profile and by reducing SARS-CoV-2 viral load and the likelihood of COVID-19 infection.

In conclusion, we propose a model for how SARS-CoV2 can potentially shape CNS autoimmunity *via* parallel activity in the CNS microenvironment and immune periphery. Further research is warranted in several key areas before a more conclusive link between SARS-CoV-2 and MS can be made.

## Data Availability Statement

The original contributions presented in the study are included in the article/supplementary material. Further inquiries can be directed to the corresponding authors.

## Author Contributions

MM and JEHS wrote the manuscript. PB and MR supervised the project and helped write and edit the manuscript. All authors contributed to the article and approved the submitted version.

## Funding

MM was supported by a Fulbright Canada-MITACS Globalink Studentship. MR holds a Junior-2 salary support award from the Fonds de recherche du Québec-Santé.

## Conflict of Interest

The authors declare that the research was conducted in the absence of any commercial or financial relationships that could be construed as a potential conflict of interest.

## Publisher’s Note

All claims expressed in this article are solely those of the authors and do not necessarily represent those of their affiliated organizations, or those of the publisher, the editors and the reviewers. Any product that may be evaluated in this article, or claim that may be made by its manufacturer, is not guaranteed or endorsed by the publisher.
